# Randomized Trial Evaluating a Self-Guided Lifestyle Intervention Delivered via Evidence-Based Materials versus a Waitlist Group on Changes in Body Weight, Diet Quality, Physical Activity, and Quality of Life among Breast Cancer Survivors

**DOI:** 10.3390/cancers15194719

**Published:** 2023-09-25

**Authors:** Leah S. Puklin, Maura Harrigan, Brenda Cartmel, Tara Sanft, Linda Gottlieb, Bin Zhou, Leah M. Ferrucci, Fang-Yong Li, Donna Spiegelman, Mona Sharifi, Melinda L. Irwin

**Affiliations:** 1 Yale School of Public Health, Yale University, New Haven, CT 06510, USA; leah.puklin@yale.edu (L.S.P.); leah.ferrucci@yale.edu (L.M.F.); fang-yong.li@yale.edu (F.-Y.L.);; 2 Yale Cancer Center, New Haven, CT 06510, USA; 3 Yale School of Medicine, Yale University, New Haven, CT 06510, USA

**Keywords:** obesity, weight loss program, lifestyle intervention, diet quality, nutrition, physical activity, exercise, breast cancer, survivorship

## Abstract

**Simple Summary:**

While resource-intensive lifestyle interventions for breast cancer survivors have proved effective at stimulating positive behavior change and promoting healthy weight loss, integrating these programs into clinical practice is challenging. To address these challenges, we adapted our supervised in-person/telephone Lifestyle, Exercise and Nutrition (LEAN) lifestyle program for breast cancer survivors with overweight or obesity to a 6-month, unsupervised, self-guided program, delivered via printed materials and online videos. We tested the efficacy of the LEAN Self-Guided program on weight loss, diet quality, physical activity, and quality of life. At 6 months, the intervention arm had significantly greater weight loss compared with the waitlist group (mean difference = −1.3 kg, 95% confidence interval [CI] = −2.5, −0.13) and maintained this weight loss from 6 months to 12 months (−0.21 kg; *p* = 0.75). Low-resource-intensive programs have the potential to be delivered in diverse healthcare settings and may support breast cancer survivors in achieving a healthy body weight.

**Abstract:**

**Background:** Lifestyle interventions for breast cancer survivors have proved effective at stimulating positive behavior change and promoting healthy weight loss, although integrating these programs into clinical practice is challenging. We evaluated the effect of a 6-month, unsupervised, self-guided, lifestyle intervention using printed materials and online videos vs. waitlist group on body weight for breast cancer survivors. **Methods:** The Lifestyle, Exercise and Nutrition (LEAN) Self-Guided trial randomized breast cancer survivors with a body mass index ≥25 kg/m^2^ to a 6-month lifestyle intervention (N = 102) or waitlist group (N = 103). Effects of the intervention on self-reported body weight, physical activity (PA), diet quality (via Health Eating Index—2010 (HEI-2010)), and quality of life were assessed using mixed model repeated measures analysis. **Results:** At 6 months, the intervention arm had significantly greater weight loss compared with the waitlist group (mean difference = −1.3 kg, 95% confidence interval [CI] = −2.5, −0.13). We observed suggestive improvements in PA (mean difference = 18.7 min/week, 95% CI = −24.2, 61.6), diet quality (mean difference in HEI = 3.2 points, 95% CI = −0.20, 6.5), and fatigue (mean difference in Functional Assessment of Chronic Illness Therapy—Fatigue scale = 1.4 points, 95% CI = −1.1, 3.9). **Conclusions:** The LEAN Self-Guided intervention led to favorable weight changes over 6 months. Low-resource-intensive programs have the potential to be delivered in diverse healthcare settings and may support breast cancer survivors in achieving a healthy body weight.

## 1. Introduction

Obesity at breast cancer diagnosis is associated with a higher risk of recurrence, breast cancer-specific mortality, and all-cause mortality [1,2]. Over 62% of breast cancer survivors are overweight (body mass index (BMI) ≥ 25 kg/m^2^) or have obesity (BMI ≥ 30 kg/m^2^) at diagnosis, and the annual increase in obesity prevalence among breast cancer survivors is one of the highest among all cancer survivors [3,4]. Additionally, every 5 kg/m^2^ increase in BMI following diagnosis is associated with a 29% higher risk of breast cancer-specific mortality [2].

Given obesity’s adverse impact at diagnosis and throughout breast cancer survivorship on health outcomes, lifestyle recommendations for cancer survivors focus on improving diet quality, promoting physical activity, and attaining a healthy weight [3,5,6,7,8]. The American Cancer Society’s 2022 updated guidelines for breast cancer survivorship included following a dietary pattern rich in vegetables, fruits, and whole grains, and engaging in 150 min of weekly aerobic exercise and twice-weekly resistance training [8]. Observational data indicate that adhering to cancer survivorship lifestyle recommendations can improve survival outcomes [9,10,11,12,13]. Among 7088 women with breast cancer, those with the highest adherence to the survivorship guidelines had a 24% lower risk of breast cancer-specific mortality and 37% lower risk of all-cause mortality compared to those with the lowest adherence [9]. Randomized lifestyle trials for breast cancer survivors have also shown that improving diet quality and increasing physical activity not only reduce treatment-related side effects, but also improve overall quality of life (QOL), body composition, serum inflammatory and metabolic biomarkers, and tumor tissue biomarkers [14,15,16,17,18,19].

Despite the benefits of lifestyle interventions, integrating them into clinical practice has proven challenging due to system-level barriers, including high intervention costs and limited personnel to deliver supervised programs [20]. However, as the population of cancer survivors grows, the demand for these services is increasing. In a survey of 531 breast cancer patients, 56% expressed interest in receiving both diet-related and exercise programs, with a majority preferring mailed literature or videos over telephone counseling [21]. Therefore, it is crucial to develop effective programs that align with patient preferences and are scalable for disseminating across healthcare settings. 

The Lifestyle, Exercise and Nutrition (LEAN) study was a randomized trial comparing in-person vs. telephone weight loss counseling vs. usual care among breast cancer survivors with overweight or obesity. LEAN resulted in significant weight loss over 6 months for in-person counseling (−6.4%) and telephone-based counseling (−5.4%) compared with usual care (−2.0%) [15]. However, to reduce intervention resources, align with patient preferences, and facilitate widespread dissemination, we adapted the LEAN intervention to an unsupervised, self-guided, lifestyle program using an evidence-based 26-chapter book, journal, and online videos. Here we evaluated the efficacy of the LEAN Self-Guided lifestyle intervention vs. a waitlist group on the primary outcome of weight at 6 months, as well as our secondary outcomes of diet quality, physical activity, and QOL at 6 months and weight at 12 months.

## 2. Materials and Methods

### 2.1. Study Participants and Recruitment

Eligible participants were breast cancer survivors <75 years of age, diagnosed with Stage 0-IIIC, with a BMI ≥ 25.0 kg/m^2^ who had completed chemotherapy and/or radiation therapy. Women had to be physically able to exercise, agree to be randomly assigned to either arm, provide informed consent, accessible by telephone, and be able to read and communicate in English. Women were ineligible if they were pregnant or intending to become pregnant in the next year, had a stroke or myocardial infarction in the past 6 months, or had a severe uncontrolled mental illness. The Yale School of Medicine Human Investigation Committee approved all procedures, including written informed consent.

Breast cancer survivors were identified and recruited from 28 July 2016 through 17 February 2017 using four approaches: (1) the Smilow Cancer Hospital at Yale-New Haven Tumor Registry; (2) direct MD referrals from Smilow Cancer Hospital and Smilow Survivorship Clinic; (3) self-referrals via clinicaltrials.gov; and (4) women previously ineligible for our prior studies.

### 2.2. Design and Randomization

Interested and eligible breast cancer survivors were mailed a baseline packet containing paper questionnaires (with an online link for those who preferred) on body weight, physical activity, diet quality, quality of life, sociodemographic characteristics, and medical history, and a standard digital scale to monitor weight (Taylor Corporation: Compact Travel Digital Personal Glass Scale). Questionnaires had to be completed prior to randomization. Participants were randomized to either the intervention or waitlist group with equal probability (1:1). Lists were generated by the study statistician using blocked randomization with varying block sizes of 4 and 6, and sealed envelopes were prepared for allocating participants. Women were notified of group assignment via telephone by the study staff.

Participants randomized to intervention were mailed a packet consisting of an introductory instructional letter; the LEAN Self-Guided book and journal; access to the LEAN Self-Guided videos on our study website; and a pedometer to track steps. Participants randomized to the waitlist group received standard medical care. At 6 months, both intervention and waitlist participants completed questionnaires similar to those at baseline. There was no contact with study participants in either the intervention or the control groups, other than at the baseline and 6-month assessments. Once the completed 6-month questionnaires were received, women in the waitlist group were mailed the LEAN Self-Guided book and journal, a pedometer, and given access to the online videos. No additional materials were provided to the intervention arm at 6 months. Follow-up 12-month weight data were assessed via questionnaire. 

### 2.3. Weight Loss Intervention

The LEAN Self-Guided intervention was derived from the original LEAN in-person/telephone trial [15]. Feedback from two focus groups, including 18 participants previously enrolled in the intervention or the usual care arm of the LEAN in-person/telephone trial, guided the adaptation process. These qualitative data were used to modify the intervention materials, including expanding the 11-session LEAN intervention book into the 26-session book (one chapter per week over 6 months; Table of Contents in Appendix A.1) and developing a LEAN website with instructional and motivational videos. 

The LEAN Self-Guided book and videos provided guidance on increasing fruit and vegetable servings, reducing energy intake (1200 to 2000 kcal/day), increasing fiber, and limiting dietary fat (<25% of total energy). The home-based physical activity goal was to accumulate 150 min per week of brisk walking (or other moderate intensity activity of choice). Pedometers were provided to track steps, with the aim of achieving 10,000 steps per day and reducing sedentary time. Participants were instructed to weigh themselves unclothed at the same time and day each week using the study-provided scale. The LEAN Self-Guided book and journal also covered behavior change strategies (e.g., self-monitoring and goal setting), with content based on the Social Cognitive Theory.

#### 2.3.1. Primary Outcome—Body Weight

Body weight was self-reported at baseline, 6 months, and 12 months via mailed or online questionnaires. Some individuals were contacted by telephone for these data if they did not return the follow up questionnaires.

#### 2.3.2. Secondary Outcomes

Physical activity was assessed at baseline and 6 months via mailed or online questionnaires. The questionnaire assessed the past 6 months of physical activity, including hours/week spent participating in different types (recreational, household, and occupation) and intensities (light, moderate, and vigorous intensity) of activity [22]. Minutes per week of moderate-to-vigorous exercise were calculated from the questionnaire.

Dietary intake was assessed at baseline and 6 months with a mailed 120-item food frequency questionnaire (FFQ) [23]. The Healthy Eating Index Score—2010 (HEI-2010) was calculated as a measure of diet quality and ranges from 0–100, with a higher score indicating better diet quality. We also assessed the following dietary components separately as these were addressed by our intervention: fruits (servings/day), vegetables (servings/day), fiber (g/1000 kcal), and % dietary fat.

Self-reported QOL was collected at baseline and 6 months using several of the Functional Assessment of Cancer Therapy (FACT) questionnaires, with higher scores indicating higher QOL. The FACT—General (FACT-G) is a 27-item questionnaire assessing physical well-being, social/family well-being, emotional well-being, and functional well-being (range from 0–108) [24]. The FACT-B (for breast cancer patients) questionnaire consists of 36 items which include the FACT-G, as well as 9 additional concerns for women with breast cancer (range from 0–144) [25,26]. The FACT—Endocrine Symptoms (FACT-ES) is a subscale including 19 items related to symptoms such as hot flashes and night sweats (range from 0–184) [27]. The Functional Assessment of Chronic Illness Therapy—Fatigue (FACIT-F) questionnaire assessed changes in fatigue over the study period (range from 0–52) [28].

### 2.4. Statistical Analysis

Baseline characteristics were compared by randomization arm using Student’s *t*-tests for continuous variables and chi-squared tests or Fischer’s exact test for categorical variables.

Mean baseline to 6-month changes were compared by randomization arms using a mixed model repeated measures analysis as intention to treat (ITT). Percent weight change from baseline to 6 months was also calculated. Linear contrasts were used to obtain changes in body weight, physical activity, dietary intake, and QOL in each group and group differences. Least square means and 95% confidence intervals (CIs) estimated from the models were reported. We explored potential effect modification on 6-month changes in body weight by BMI, age, disease stage, education, employment status, marital status, menopausal status, receipt of chemotherapy, receipt of radiation, time since diagnosis, and FACT-B by including a group by time by modifier interaction term in the model. 

We explored 6-month to 12-month changes in weight for each arm independently, as well as percent weight change. For the women in the intervention arm, this time period was an extended follow-up with no further intervention materials, and for the waitlist group, this was the time period in which they received the intervention materials.

We investigated differential losses to follow-up by study arm at 6 months and performed two sensitivity analyses: adjusting for baseline factors associated with drop-out; and repeated measures ANCOVA with only complete data. 

All analyses were conducted using SAS Version 9.4 (SAS, Cary, NC, USA) and statistical tests were two sided, with a 0.05 statistical significance level.

## 3. Results

**Figure 1:**
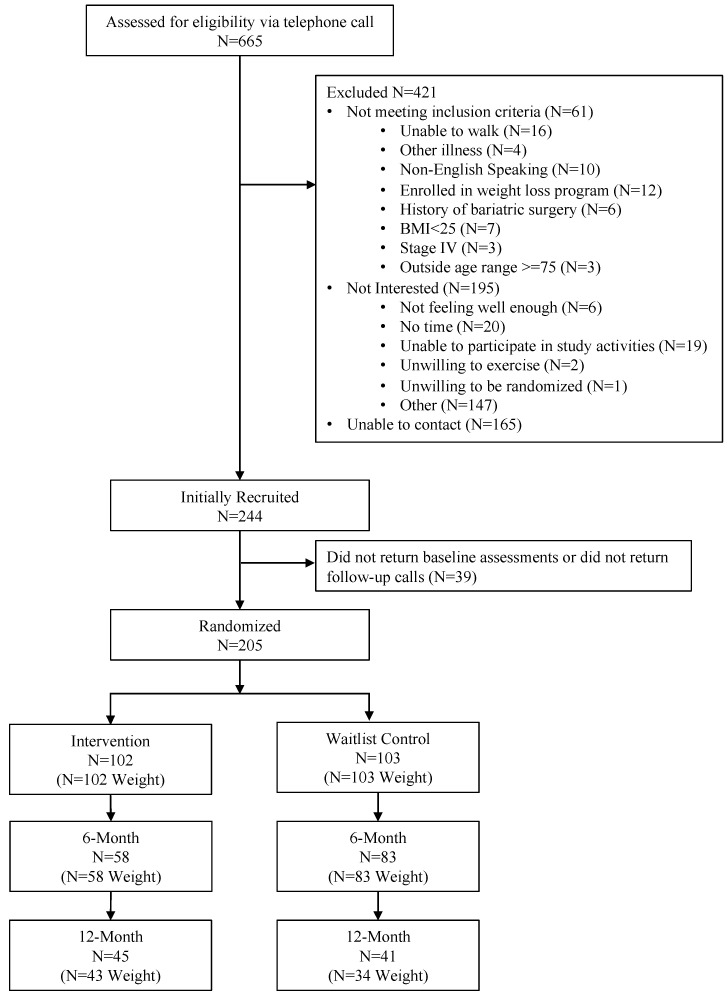
Consort diagram.

We identified 665 breast cancer survivors who we attempted to call to assess eligibility. A total of 205 women were eligible to be randomized after 61 were found to be ineligible, 165 were unable to be contacted, and 195 were not interested (Figure 1). 

### 3.1. Baseline Characteristics

Baseline characteristics were similar by study arm (Table 1). On average, women were 57.4 ± 10.4 years of age, 3.7 ± 3.6 years from diagnosis, and had a BMI of 32.3 ± 5.0 kg/m^2^. The majority of women were married or living with a partner (68.3%), had at least a college education (83.4%), were non-Hispanic White (86.3%), were employed full-time (48.8%), had stage I disease (43.6%), received chemotherapy treatment (53.2%), and were postmenopausal (86.8%).

### 3.2. Body Weight at 6-Months

Average 6-month weight loss was −2.1 kg (2.4%) (*p* < 0.001) and −0.73 kg (0.85%) (*p* = 0.07) in the intervention and waitlist groups, respectively, with a statistically significant effect size (mean difference = −1.3 kg, 95% CI = −2.5, −0.13; *p* = 0.03) (Table 2).

At 6 months, we observed differential losses to follow-up by study arm, with 58 (58%) women completing assessments in the intervention arm compared with 83 (81%) in the waitlist group (*p* < 0.001). Age, race and ethnicity, baseline minutes per week of exercise, baseline HEI score, and baseline fiber intake were associated with loss to follow-up, but our sensitivity analyses for 6-month weight change adjusted for these factors and models using only complete data did not differ from our primary model.

Only menopausal status was a significant effect modifier of 6-month body weight change (*p* = 0.03) (Table 3). Among postmenopausal women, there was no significant difference in weight loss by study arm (*p* = 0.06). Premenopausal women in the intervention arm lost a mean of 1.1 kg (standard error [SE] = 1.1) vs. a gain of 4.1 kg (SE = 1.4) among those in the waitlist group (*p* = 0.003).

### 3.3. Changes in Secondary Outcomes at 6-Months

Change in physical activity from baseline to 6 months did not significantly differ between arms, with the intervention group increasing 30.8 min/week (95% CI = −1.7, 63.3) compared to the waitlist group increasing 12.1 min/week (95% CI = −16.0, 40.1) (mean difference = 18.7 min/week, 95% CI = −24.2, 61.6; *p* = 0.39) (Table 4). Over 6 months, there was a suggestive improvement in HEI-2010, with the intervention group improving diet quality (measured by HEI) by 4.6 points (95% CI = 2.0, 7.3) compared to the waitlist control group improving diet quality (measured by HEI) by 1.4 points (95% CI = −0.6, 3.5) (mean difference = 3.2 points, 95% CI = −0.20, 6.5; *p* = 0.07). Women in the intervention arm increased their servings of vegetables by 0.54 servings/day (95% CI = 0.16, 0.91) compared to the waitlist group who decreased their consumption of vegetables by 0.13 servings/day (95% CI = −0.41, 0.15) (mean difference = 0.67 servings/day, 95% CI = 0.20, 1.13; *p* = 0.01). No significant intervention effects were observed for FACT-G, FACT-B, or FACT-ES. Over 6 months, there was a suggestive improvement in the FACIT-F scale among the intervention group (2.7 points, 95% CI = 0.77, 4.7) compared to the waitlist group (1.3 points, 95% CI = −0.28, 2.8) (mean difference = 1.4 points, 95% CI = −1.1, 3.9; *p* = 0.26). 

### 3.4. Twelve-Month Follow-Up

At 12 months, a total of 77 participants provided weight measurements (intervention N = 43, waitlist group N = 34) (Figure 1). Over the 6-month extended follow-up period following the intervention, women randomized to the original intervention arm experienced weight maintenance (−0.21 kg from 6 months to 12 months (−0.21%); *p* = 0.75) (Table 2). At 6 months, the waitlist group received the intervention materials. During the delayed intervention period, from 6 months to 12 months, women in the waitlist group lost weight (−2.9 kg (−3.4%); *p* < 0.001).

## 4. Discussion

The LEAN Self-Guided lifestyle program delivered via printed and online materials led to statistically significant weight loss at 6 months for breast cancer survivors with a BMI ≥ 25 kg/m^2^ (−2.1 kg (−2.4%) versus −0.73 kg (−0.85%), mean difference = −1.3 kg; *p* = 0.03). We observed suggestive improvements in PA (mean difference = 18.7 min/week, 95% CI = −24.2, 61.6), diet quality (mean difference in HEI = 3.2 points, 95% CI = −0.20, 6.5), and fatigue (mean difference in FACIT-F scale = 1.4 points, 95% CI = −1.1, 3.9). Women randomized to the original intervention arm were able to successfully maintain weight loss 6 months after the end of the intervention (−0.21 kg from 6 months to 12 months; *p* = 0.75).

The weight loss observed was slightly lower than that of the original LEAN telephone/in-person trial from which LEAN Self-Guided materials were adapted and was less than the clinically meaningful threshold of 5% body weight change [29,30,31]. The original 6-month LEAN telephone/in-person trial of 11 counseling sessions led by a registered dietitian resulted in a 6.4% weight loss in the in-person arm, 5.4% loss in the telephone arm, and 2.0% loss in the usual care arm. The attenuated effect observed in our current study may be partially explained by the unsupervised nature of the LEAN Self-Guided trial, which only included one-time mailed materials [15]. According to a recent meta-analysis, professional monitoring through counseling or check-ins was frequently cited as a key facilitator of adherence to nutrition, physical activity, and lifestyle behavioral interventions [32]. Supervised interventions offer psychological benefits, such as positive reinforcement and increased accountability, leading to improved adherence [33,34]. However, supervised interventions tend to be costly and time intensive, and may not be feasible in many settings, including community settings where most cancer care is delivered [35]. A cost-effectiveness analysis of the Exercise for Health intervention, an 8-month telephone-delivered aerobic and resistance exercise intervention involving 16 sessions with an exercise physiologist, found personnel costs were approximately AUD 758.53 per person (~USD 487.64), whereas the intervention materials (educational workbook and exercise tracker) cost only AUD 9.40 per person (~USD 6.04) [36]. 

Limited research has examined the effect of self-guided lifestyle interventions for cancer patients using solely printed or online materials, and to our knowledge, no prior studies have been tailored specifically for weight loss among breast cancer survivors with overweight or obesity. The FRESH START trial enrolled 543 newly diagnosed breast and prostate cancer patients and compared a 10-month program delivered through tailored workbooks and mailed newsletters to standard mailed print materials on diet quality and exercise [37]. Similar to our study, they did not find significant changes in QOL measured by the FACT-G. However, they did observe increases in weekly exercise for the intervention compared to attention control (+59.3 vs. +39.2 min/week, *p* = 0.02). In contrast to our null exercise findings, these improvements in exercise may be partially explained by their exclusion of individuals already practicing healthy behaviors and more frequent contact with participants (every 6 weeks) [37]. Another four-arm trial involving 377 breast cancer survivors compared (1) breast-cancer-specific physical activity printed materials; (2) a step pedometer; (3) a combination of printed materials and a pedometer; and (4) standard public health recommendations. The trial found a significant increase in minutes/week of exercise for the combined materials group compared to the standard recommendation (+87 vs. +30 min/week, *p* = 0.022) [38]. However, the exercise findings are difficult to compare to ours as they used a different questionnaire (leisure score index vs. modifiable physical activity questionnaire). They also noted significant improvements in QOL and fatigue [38]. Lastly, a three-arm trial in 173 breast cancer survivors randomized women to (1) bi-weekly mailed information similar to the FRESH START trial; (2) bi-weekly mailed information with additional materials to develop skills, create awareness, and self-reflection; or (3) usual care including standardized lifestyle management information [39]. This study found modest, short-term positive effects on fruit and vegetable consumption among women receiving the additional materials [39]. Consistent with these findings, we observed a borderline significant effect on diet quality over 6 months, and since self-report dietary data are subject to non-differential measurement error, this could have attenuated our results toward the null. 

Menopausal status was a significant modifier of the intervention on weight loss, with premenopausal women having the greater benefit from the intervention (*p* = 0.03). Specifically, our study found that premenopausal women in the waitlist control group gained 4.1 kg over 6 months compared to a loss of 1.1 kg among the intervention group (*p* = 0.003). These findings align with the current literature that suggests premenopausal status is associated with post-diagnosis weight gain [40,41]. Chemotherapy-associated amenorrhea, as well as ovarian-suppressing drugs, are considered the main drivers of weight gain among this population [42,43,44]. The adverse effect of weight and weight gain on survival is stronger among premenopausal women with breast cancer compared to postmenopausal women [45]. However, our observation of a stronger effect of the LEAN Self-Guided intervention in premenopausal women should be interpreted with caution given the small sample size of premenopausal women in our study (14%). We hypothesize that the greater flexibility of a self-directed lifestyle program may been ideal for younger breast cancer survivors who experience greater competing priorities, such as family caregiving roles [46]. These findings, if replicated in other studies, could be useful in tailoring future interventions.

While many short-term lifestyle interventions for breast cancer survivors have resulted in weight loss during the intervention period, a couple of studies have reported weight regain following the intervention period [47,48]. However, during our 6-month extended follow-up period, the intervention arm sustained their weight loss (−0.21 kg (−0.25%)). Similarly, in a study on the long-term follow-up of the LEAN in-person/telephone study participants, both intervention arms maintained their original intervention-period weight loss [49]. Surprisingly, in our trial, the waitlist group showed greater weight loss during the delayed intervention period (−2.9 kg (−3.4%), 6 months to 12 months) compared with the intervention arm during the original 6-month intervention period (−0.21 kg (−2.5%), baseline to 6 months). This could be due to the waitlist group’s high motivation upon receiving the LEAN Self-Guided program, which was also demonstrated by their higher response rate at 6 months (81% vs. 58% in the intervention arm, *p* < 0.001). 

It is also important to consider that the LEAN Self-Guided program was delivered to women who were, on average, 3.7 years post-diagnosis and were primarily diagnosed with early-stage disease. Women who are in active treatment may benefit from interventions with greater contact with study personnel. Similarly, women who are diagnosed with advanced stage disease may require supervised and tailored interventions to address their greater disease burden and higher prevalence of cachexia. However, our results provide evidence that a low-resource-intensive program may be an effective option for early-stage women who are out of active treatment. Lifestyle interventions for breast cancer survivors are not a one-size-fits all approach. The LEAN Self-Guided content could be used within survivorship care where patients are referred to different lifestyle interventions of varying doses, levels of supervision, and delivery modes based on the patients’ needs and preferences.

Recognizing the importance of body composition measures (i.e., adipose vs. muscle) in addition to body weight and BMI is critical for fully characterizing the effect of lifestyle interventions and the impact on disease risk, recurrence, and mortality. Future studies should measure and validate novel remote assessment methods for body composition. Our most recent trial, the Lifestyle, Exercise and Nutrition Early After Diagnosis (LEANer) study focused on weight management during chemotherapy and the impact on chemotherapy completion rates, and will investigate body composition changes during the year-long intervention [50].

Our study had several strengths, including the use of validated questionnaires for dietary intake, physical activity, and QOL. The adaptation of materials based on feedback from breast cancer survivors aligned our materials with patient preferences and needs. The LEAN Self-Guided trial required limited resources and use of professional personnel, making this an easily scalable program. Our study also had some limitations. We relied on self-reported weight, which may have introduced social-desirability bias. We also used self-report measures of exercise, diet quality, and quality of life, rather than objective measures, which may, in part, explain the larger observed standard deviations and the non-significant effect sizes. With more precise assessment methods and a larger sample size that would increase statistical power to detect differences for these secondary outcomes, it is possible we would have observed smaller standard deviations and statistically significant effects of our intervention. We also observed differential losses to follow-up by study arm, although reasons captured for discontinuation of participation were explored during follow-up calls with both arms and reasons were found to be likely unrelated to randomization (i.e., unrelated surgery and/or illnesses, family concerns). Our intervention did not include any contact with participants regardless of study arm over the 6 months. The limited contact intervention and the waitlist control design may have created a stronger motivation and incentive for the waitlist control group to comply at 6 months. Future studies that incorporate mhealth or distance-based monitoring may lead to higher compliance with both groups. Lastly, our sample primarily consisted of non-Hispanic white women with a high level of education, which many limit the generalizability of our findings to populations disproportionately affected by obesity and health inequities.

## 5. Conclusions

The LEAN Self-Guided lifestyle intervention resulted in weight loss at 6 months among women who had completed breast cancer treatment and had a BMI ≥ 25 kg/m^2^. Future research should explore the optimal dose, level of supervision, and delivery mode of lifestyle interventions for this population to support survivorship care referral systems that meets patients’ needs and preferences. However, low-resource-intensive programs have the potential to be implemented in diverse healthcare settings and play an important role in supporting breast cancer survivors in achieving a healthy body weight.

## Figures and Tables

**Table 1:** Baseline characteristics by randomization arm (N = 205).

Characteristic	Total Population ^a,b^ N = 205	Intervention ^a,b^ N = 102	Waitlist Group ^a,b^ N = 103	*p*-Value ^c^
**Age (years)**	57.4 ± 10.4	57.0 ± 10.7	57.9 ± 10.0	0.54
**Marital Status**				0.58
Married or Living with Partner	140 (68.3)	69 (67.7)	71 (68.9)	
Divorced or Separated/Never Married/Widowed	64 (31.2)	33 (32.4)	31 (30.1)	
Prefer not to answer	1 (1.0)	0 (0.0)	1 (1.0)	
**Education**				0.60
College and above	171 (83.4)	85 (83.3)	86 (83.5)	
Less than College	33 (16.1)	17 (16.7)	16 (15.5)	
Prefer not to answer	1 (0.5)	0 (0)	1 (1.0)	
**Race and Ethnicity**				0.08
Non-Hispanic White	177 (86.3)	84 (82.4)	93 (90.3)	
Non-Hispanic Black	16 (7.8)	13 (12.8)	3 (2.9)	
Hispanic	6 (2.9)	3 (2.9)	3 (2.9)	
Asian/Pacific Islander	1 (0.5)	0 (0.0)	1 (1.0)	
Prefer not to answer	1 (0.5)	1 (1.0)	0 (0.0)	
Other ^d^	4 (2.0)	1 (1.0)	3 (2.9)	
**Employment**				0.74
Full Time (≥35 h/wk)	100 (48.8)	48 (47.1)	52 (50.5)	
Part time (<35 h/wk)	35 (17.1)	16 (15.7)	18 (17.5)	
Unemployed/Retired	67 (32.7)	36 (35.3)	31 (30.1)	
Prefer not to answer	1 (0.5)	0 (0.0)	1 (1.0)	
Other ^e^	2 (1.0)	2 (2.0)	1 (1.0)	
**Weight (kg)**	85.6 ± 14.8	85.6 ± 13.9	85.6 ± 15.7	0.98
**BMI (kg/m^2^)**	32.3 ± 5.0	32.3 ± 4.8	32.2 ± 5.3	0.88
**Postmenopausal**	177 (86.8)	86 (85.2)	91 (88.4)	0.42
**Time since diagnosis (years)**	3.7 ± 3.6	3.6 ± 3.1	3.8 ± 4.0	0.70
**Recurrence before randomization**	11 (5.5)	5 (5.0)	6 (6.0)	0.74
**Cancer Stage**				0.37
0	27 (13.9)	11 (11.6)	16 (16.0)	
I	85 (43.6)	45 (47.4)	40 (40.0)	
II	61 (31.3)	26 (27.4)	35 (35.0)	
III	22 (11.3)	13 (13.7)	9 (9.0)	
**Radiotherapy**	151 (42.1)	74 (74.0)	77 (76.2)	0.71
**Chemotherapy**	107 (53.2)	54 (54.0)	53 (54.3)	0.83
**Surgery**	199 (98.0)	98 (97.0)	101 (99.0)	0.31
**Exercise (min/week)**	94.0 ± 133.3	82.0 ± 118.5	106.1 ± 146.4	0.20
**Healthy Eating Index (HEI)**	66.5 ± 11.7	65.0 ± 12.4	68.0 ± 10.9	0.07
**Fiber intake (g)**	18.3 ± 8.9	17.4 ± 7.9	19.2 ± 9.7	0.17
**Fruit (servings/day)**	1.3 ± 1.2	1.2 ± 1.2	1.4 ± 1.2	0.44
**Vegetable (servings/day)**	2.3 ± 1.5	2.2 ± 1.4	2.4 ± 1.5	0.30
**% Fat**	34.9 ± 6.7	35.4 ± 7.1	34.4 ± 6.2	0.30
**FACT-G**	84.9 ± 15.0	84.8 ± 15.4	85.1 ± 14.8	0.91
**FACT-B**	107.0 ± 19.1	106.7 ± 19.8	107.2 ± 18.5	0.84
**FACT-ES**	55.6 ± 11.2	54.3 ± 11.5	56.9 ± 10.7	0.11
**FACIT-F**	37.4 ± 10.5	36.9 ± 9.6	37.8 ± 11.4	0.54

Abbreviations: BMI, body mass index; FACT-G, functional assessment of cancer therapy general; FACT-B, functional assessment of cancer therapy breast cancer; FACT-ES, functional assessment of cancer therapy endocrine symptoms; FACIT-F, functional assessment of chronic illness therapy-fatigue. ^a^ Mean ± standard deviation for continuous variables and n (column %) for categorical variables. ^b^ Numbers may not sum to the total due to missing and percentages may not sum to 100% due to rounding. ^c^ *p* value is for *t*-test (continuous variables), chi-square test (categorical variables), or Fisher’s exact test (cell counts < 5). ^d^ N = 1 Jamaican, N = 1 Italian, N = 1 Black/African American and American Indian or Alaskan Native, N = 1 Cape Verde. ^e^ N = 1 Graduate student, N = 1 Substitute Teacher, N = 1 Per Diem.

**Table 2:** Effect of LEAN intervention versus waitlist groups on body weight changes at 6 months (N = 205) and within group changes from 6 months to 12 months (N = 141).

Time Period	Intervention ^a^ N = 102	Waitlist Group ^a^ N = 103	Effect Size, Least Square Mean (95% CI) ^a^	*p*-Value ^a^
**Baseline to 6-Month Period**				
Common baseline weight (kg)	85.6 (83.6, 87.6)			
Weight change (kg)	−2.1 (−3.0, −1.1)	−0.73 (−1.5, 0.05)	−1.3 (−2.5, −0.13)	0.03
*p* value ^b^	<0.001	0.07		
**6-Month to 12-Month Follow-up Period** (*Intervention Only*)	**Intervention** **N = 58**	**Waitlist Group** **N = 83**		
Weight change (kg)	−0.21 (−1.5, 1.1)	--	--	
*p* value ^b^	0.75	--	--	
**6-Month to 12-Month Delayed Intervention Period** (*Waitlist Group Only*)	**Intervention** **N = 58**	**Waitlist Group** **N = 83**		
Weight change (kg)	--	−2.9 (−4.3, −1.5)	--	
*p* value ^b^	--	<0.001	--	

^a^ Mixed effect model; ^b^ Within group differences.

**Table 3:** Intervention effect on body weight stratified by baseline demographic and clinical characteristics.

6-Month Change	N		Weight (kg)				
	Intervention	Waitlist Group	Intervention LSmean (SE) ^a^	Waitlist Group LSmean (SE) ^a^	Effect Size (95% CI)	*p*	*P_interaction_*
**BMI (kg/m^2^)**							0.18
≥30	67	61	−2.1 (0.61)	−0.48 (0.51)	−1.7 (−3.2, −0.10)	0.04	
<30	35	42	0.14 (0.71)	0.18 (0.59)	−0.04 (−1.8, 1.8)	0.97	
**Age, years (median)**							0.42
≥57	53	56	−2.3 (0.55)	−0.68 (0.49)	−1.7 (−3.1, −0.23)	0.02	
<57	49	47	−0.27 (0.67)	0.47 (0.57)	−0.74 (−2.5, 0.99)	0.40	
**Clinical Stage**							0.31
0/I	56	56	−2.6 (0.60)	−0.50 (0.49)	−2.1 (−3.6, −0.52)	0.01	
II/III	39	44	−1.0 (0.71)	−0.18 (0.60)	−0.84 (−2.7, 0.98)	0.37	
**Education**							0.47
College or above	85	86	−1.6 (0.50)	−0.46 (0.41)	−1.2 (−2.4, 0.12)	0.07	
Below college	17	16	−1.4 (0.91)	0.82 (1.01)	−2.2 (−4.9, 0.42)	0.10	
**Employed**							0.19
Employed	64	70	−0.79 (0.57)	−0.15 (0.46)	−0.64 (−2.1, 0.81)	0.38	
Not Employed	36	31	−2.4 (0.70)	−0.20 (0.66)	−2.2 (−4.1, −0.32)	0.02	
**Marriage**							0.83
Married/Living with someone	69	71	−1.7 (0.54)	−0.26 (0.45)	−1.4 (−2.8, −0.01)	0.05	
Live Alone	33	31	−1.4 (0.76)	−0.23 (0.72)	−1.1 (−3.2, 0.94)	0.28	
**Menopausal Status**							0.03
Postmenopausal	86	91	−1.7 (0.46)	−0.58 (0.38)	−1.1 (−2.3, 0.06)	0.06	
Premenopausal	15	11	−1.1 (1.1)	4.1 (1.4)	−5.1 (−8.6, −1.7)	0.003	
**Chemotherapy**							0.45
Yes	54	53	−1.0 (0.60)	−0.01 (0.51)	−1.0 (−2.6, 0.52)	0.19	
No	46	48	−2.4 (0.62)	−0.49 (0.55)	−1.9 (−3.5, −0.28)	0.02	
**Radiation**							0.72
Yes	74	77	−1.8 (0.50)	−0.24 (0.43)	−1.6 (−2.9, −0.30)	0.02	
No	26	24	−1.2 (0.91)	−0.10 (0.82)	−1.1 (−3.5, 1.3)	0.37	
**Time Since Diagnosis, years (median)**							0.80
≥2.75	54	47	−2.0 (0.55)	−0.74 (0.56)	−1.3 (−2.8, 0.27)	0.11	
<2.75	48	56	−0.80 (0.70)	0.18 (0.50)	−0.98 (−2.7, 0.71)	0.25	
**Baseline FACT-B (median)**							0.18
≥109	51	53	−2.5 (0.59)	−0.46 (0.51)	−2.0 (−3.5, 0.49)	0.01	
<109	51	50	−0.52 (0.64)	−0.03 (0.54)	−0.49 (−2.1, 1.2)	0.56	

Abbreviations: BMI, body mass index; FACT-B, functional assessment of cancer therapy breast cancer. Table values are estimated using mixed effect model. ^a^ Least square means (95% CI).

**Table 4:** Effect of LEAN intervention versus waitlist groups on secondary outcomes; changes at 6 months.

	N	Intervention ^a^	N	Waitlist Group ^a^	Effect Size, Least Square Mean (95% CI) ^a^	*p*-Value ^a^
**Weekly Exercise (min/week)**	100		99			
Combined baseline		95.7 (77.1, 114.4)				
Baseline to 6-Month change		30.8 (−1.7, 63.3)		12.1 (−16.0, 40.1)	18.7 (−24.2, 61.6)	0.39
**HEI-2010 Score**	99		103			
Combined baseline		66.5 (64.8, 68.1)				
Baseline to 6-Month change		4.6 (2.0, 7.3)		1.4 (−0.64, 3.5)	3.2 (−0.20, 6.5)	0.07
**Fiber intake (g/1000 kcal)**	99		103			
Combined baseline		18.3 (17.1, 19.5)				
Baseline to 6-Month change		1.1 (−0.66, 2.8)		0.44 (−0.86, 1.7)	0.62 (−1.5, 2.8)	0.57
**Fruit (servings/day)**	98		99			
Combined baseline		1.3 (1.1, 1.5)				
Baseline to 6-Month change		0.36 (0.02, 0.70)		0.09 (−0.17, 0.34)	0.27 (−0.15, 0.70)	0.21
**Vegetable (servings/day)**	97		99			
Combined baseline		2.3 (2.1, 2.6)				
Baseline to 6-Month change		0.54 (0.16, 0.91)		−0.13 (−0.41, 0.15)	0.67 (0.20, 1.13)	0.01
**Fat (%)**	99		103			
Combined baseline		34.9 (34.0, 35.8)				
Baseline to 6-Month change		−3.0 (−4.7, −1.2)		−1.2 (−2.6, 0.18)	−1.8 (−4.0, 0.47)	0.12
**FACT-G**	101		103			
Combined baseline		84.9 (82.8, 86.9)				
Baseline to 6-Month change		1.5 (−1.0, 4.1)		2.2 (0.12, 4.2)	−0.63 (−3.9, 2.7)	0.71
**FACT-B**	101		103			
Combined baseline		106.9 (104.2, 109.5)				
Baseline to 6-Month change		1.8 (−1.4, 5.1)		3.5 (0.93, 6.0)	−1.6 (−5.7, 2.5)	0.44
**FACT-ES**	101		103			
Combined baseline		55.6 (54.1, 57.1)				
Baseline to 6-Month change		2.3 (0.48, 4.2)		0.59 (−0.88, 2.1)	1.7 (−0.6, 4.1)	0.15
**FACIT-F**	101		103			
Combined baseline		37.4 (36.0, 38.9)				
Baseline to 6-Month change		2.7 (0.77, 4.7)		1.3 (−0.28, 2.8)	1.4 (−1.1, 3.9)	0.26

Abbreviations: HEI, health eating index; FACT-G, functional assessment of cancer therapy general; FACT-B, functional assessment of cancer therapy breast cancer; FACT-ES, functional assessment of cancer therapy endocrine symptoms; FACIT-F, functional assessment of chronic illness therapy-fatigue. ^a^ Intervention effect and corresponding *p*-values are estimated using a mixed effect model.

## Data Availability

The data presented in this study are available upon request from the corresponding author. The data are not publicly available to protect patient privacy.

## Appendix A

### Appendix A.1. LEAN Self-Guided Book Table of Contents

**Week/Section**	**Topics Covered|Page Number**
Introduction	How to read the LEAN book|12 LEAN Benefits|13 LEAN Goals|15 Dr. Tara: *Weight Gain from Treatment|*17 Dr. Tara: *Excess Body Fat and Breast Cancer|*18 Baseline Timed One Mile Walk|23
Week 1	Set Your Weight Loss Goals|27 Exercise Guidelines for Cancer Survivors|29 Dr. Tara: *What Causes Breast Cancer?|*34
Week 2	Concept of Energy Balance: *The “No More Dieting” Approach|*37 Shoe Selection and Exercise Wear Tips|41 Counting Steps Using a Pedometer|44 Dr. Tara: *Why Do I Feel so Tired?|*47
Week 3	Adopt the New American Plate|49 How to Exercise Safely|50
Week 4	Pump Up the Phytonutrients|55 Dr. Tara: *Tamoxifen|*59 Benefits of Exercise|60 Timed One Mile Walk # 2|65
Week 5	Portion Distortion: *Understanding Food Labels and Serving Sizes|*67 Make Time for Exercise|73 Dr. Tara: *Aromatase Inhibitors|*76
Week 6	Be a Fat Detective|79 Setting Your Daily Fat Gram Goal|82 Reducing Your Sedentary Behavior|91
Week 7	Be a Sugar Detective: *Adding Up the Sugar Grams|*95 Dr. Tara: Chemo-brain|98 Exercise Goal Setting|99
Week 8	Whole Grains: *The Other Carbohydrate* 105 Fiber: *Nature’s Gift to Our Bodies|*106 Add Variety—Cross Train!|111 Timed One Mile Walk # 3|113
Week 9	Alcohol|115 Enlist Support…Exercise Buddies|116 Dr. Tara: *Peripheral Neuropathy|*117
Week 10	Practicing Mindful Eating|119 Cardiovascular Disease and Cardiorespiratory Fitness|125
Week 11	Understanding Food Labels: *Sodium Content|*129 Dr. Tara: Lymphedema|131
Week 12	Vitamin and Supplement Use|135 Dr. Tara: *Exercise and Bone Maintenance|*141 Timed One Mile Walk # 4|144
Week 13	Mid-Point Self-Assessment|147 Exercise and Weight Loss Improve Breast Cancer Survival|152
Week 14	Grocery Shopping|155 Exercise and Reducing Cancer Related Fatigue|162
Week 15	Creating a Healthy Food Environment at Work|165 The Live**STRONG** at the YMCA Exercise Program|168
Week 16	Organic Foods|173 The Question of Soy Foods|174 Dr. Tara: *Yoga and Sleep|*178 Timed One Mile Walk # 5|179
Week 17	Dining Out|181 Exercise and Improved Joint Pain|182
Week 18	Food Safety and Your Immune System|191 Exercise and Immune Changes|193
Week 19	Choose to Lose|197 Certified Cancer Exercise Trainer|199
Week 20	Vacation/Travel Strategies|203 Times One Mile Walk # 6|206
Week 21	Talk Positively to Yourself|209 Benefits of Interval Training|213
Week 22	You Can Manage Stress|217 Dr. Tara: *Sexual Health|*220
Week 23	The Slippery Slope of Lifestyle Change|223 Coping with Lapses|224
Week 24	Healthy Communities|229 Physical Activity and Improved Survival|232 Times One Mile Walk # 7|234
Week 25	Your LEAN Toolkit|237 Reward Yourself|240
Week 26	Maintaining a Healthy Lifestyle|243 Continue to Challenge Yourself|246
Appendix	Strength Training|244
